# Neural Mechanisms of the Contextual Interference Effect and Parameter Similarity on Motor Learning in Older Adults: An EEG Study

**DOI:** 10.3389/fnagi.2020.00173

**Published:** 2020-06-12

**Authors:** Meysam Beik, Hamidreza Taheri, Alireza Saberi Kakhki, Majid Ghoshuni

**Affiliations:** ^1^Motor Behavior Laboratory, Department of Motor Behavior, Faculty of Sport Sciences, Ferdowsi University of Mashhad, Mashhad, Iran; ^2^Department of Biomedical Engineering, Islamic Azad University, Mashhad Branch, Mashhad, Iran

**Keywords:** contextual interference effect, EEG, motor learning, optimal error, older adults, parameter similarity

## Abstract

The purpose of this study was to investigate the neural mechanisms of the contextual interference effect (CIE) and parameter similarity on motor learning in older adults. Sixty older adults (mean age, 67.68 ± 3.95 years) were randomly assigned to one of six experimental groups: blocked-similar, algorithm-similar, random-similar, blocked-dissimilar, algorithm-dissimilar, and random-dissimilar. Algorithm practice was a hybrid practice schedule (a combination of blocked, serial, and random practice) that switching between practice schedules were based on error trial number, ≤33%. The sequential motor task was used to record the absolute timing for the absolute timing goals (ATGs). In similar conditions, the participants’ performance was near ATGs (1,350, 1,500, 1,650 ms) and in dissimilar conditions, they performed far ATGs (1,050, 1,500, 1,950 ms) with the same spatial sequence for all groups. EEG signals were continuously collected during the acquisition phase and delayed retention. Data were analyzed in different bands (alpha and beta) and scalp locations (frontal: Fp1, Fp2, F3, F4; central: C3, C4; and parietal: P3, P4) with repeated measures on the last factor. The analyses were included motor preparation and intertrial interval (motor evaluation) periods in the first six blocks and the last six blocks, respectively. The results of behavioral data indicated that algorithm practice resulted in medium error related to classic blocked and random practice during the acquisition, however, algorithm practice outperformed the classic blocked and random practice in the delayed retention test. The results of EEG data demonstrated that algorithm practice, due to optimal activity in the frontal lobe (medium alpha and beta activation at prefrontal), resulted in increased activity of sensorimotor areas (high alpha activation at C3 and P4) in older adults. Also, EEG data showed that similar conditions could affect the intertrial interval period (medium alpha and beta activation in frontal in the last six-block), while the dissimilar conditions could affect the motor preparation period (medium alpha and beta activation in frontal in the first six-block). In conclusion, algorithm practice can enhance motor learning and optimize the efficiency of brain activity, resulting in the achievement of a desirable goal in older adults.

## Introduction

Motor learning is defined as the relatively permanent changes in the capacity for movement through practice or experience (Schmidt et al., 2018, p. 283). Therefore, learning may be highly dependent on practice conditions. One of the most frequent research topics in terms of the practice conditions is about how the multiple tasks or variations of a task are arranged in a practice session. This issue is examined under the contextual interference effect (CIE). The CIE states how different orders of task performance interfere with each other to affect the outcome of practice conditions. Task orders in blocked or random formations are known as low or high contextual interference. In blocked practice, the number of the same trials is repeated before shifting to the next set of different trials, while in the random practice, the performer is faced with different types of tasks, thus there is no chance to anticipate the characteristics of the next trial. The result of several studies has shown that the random practice results in poorer performance during acquisition but superior performance during retention and transfer compared to the blocked practice (Shea and Morgan, 1979; Magill and Hall, 1990; Brady, 1998). The result of studies has also indicated that older adults may benefit from this type of practice condition like younger performers (Lin et al., 2010, 2012; Pauwels et al., 2015, 2018; Sidaway et al., 2016; Chalavi et al., 2018). However, some studies did not show the superior performance of random practice during retention in older adults (de Souza et al., 2015). In this regard, other pieces of evidence showed that variables such as age, skill level, and learning style of learners can affect the CIE (Magill and Hall, 1990; Brady, 1998, 2008; Merbah and Meulemans, 2011), and optimal learning outcome is the result of interaction between the skill level of learner and task difficulty, known as challenge point framework (Guadagnoli and Lee, 2004). Computer-controlled practice based on error rate can be used to adjust the interaction between task difficulty and progress in skill level during the acquisition phase (Choi et al., 2008; Simon et al., 2008; Wilson et al., 2018; Wadden et al., 2019). Also, task similarity is another variable that may affect the CIE (Battig, 1966; Wood and Ging, 1991; Boutin and Blandin, 2010a). Similar and dissimilar tasks are defined according to the motor program- or the distance between parameters used in the same motor program (Magill and Hall, 1990; Boutin and Blandin, 2010a). There is a contradiction between similar and dissimilar conditions concerning CIE, so that some studies showed the superior performance in dissimilar conditions of random practice (Wood and Ging, 1991; Boutin and Blandin, 2010a), whereas another study revealed the superior performance in similar conditions of random practice (Boutin and Blandin, 2010b). Likely, the similar and dissimilar conditions are not themselves the reason for the discrepancy in the findings, but creating the optimal challenge point could be the possible reason. Possibly the beneficial effect of CIE can be seen in similar and dissimilar conditions both by creating the optimal challenge point using an algorithm-based practice schedule.

Motor learning causes different neurological changes in early and late phases of learning (Galván, 2010). The results of neurological studies indicate that even in a situation that the findings of younger vs. older adults are similar, they may have a different pattern of activation in their brain (Lin et al., 2012). Some of the changes in activation patterns of the brain in older adults are related to their optimal performance because it seems that these changes are as a compensation potential and neuroplasticity in the brain of older adults (Lin et al., 2012). Also, there is some evidence that the pattern of coding and retrieval in older adults is different from that of younger people. Therefore, it is likely that there are different neurological paths in younger adults compared to older counterparts, to perform a motor task (Lin et al., 2012). Given that the CIE influences motor learning, it, therefore, can be concluded that it also affects brain activities associated with effort and motor preparation (Lage et al., 2015; Frömer et al., 2016; Thürer et al., 2017; Henz et al., 2018). Some studies have shown that CIE is associated with dorsolateral prefrontal cortex (DLPFC) and primary motor area (M1) activity (Cohen et al., 2009; Wymbs and Grafton, 2009; Kantak et al., 2010; Lin et al., 2010; Lage et al., 2015). Henz et al. (2018) examined differential learning protocol and the CIE after learning a motor skill by applying electroencephalography (EEG) technique. The result indicated that the high CIE resulted in increasing the cognitive processing activities (increased beta and gamma waves in the frontal region). EEG is a brain imaging technique that has high temporal resolution and mediates spatial resolution. In this study, concerning the task, it was needed to use brain imaging techniques with a high temporal resolution to record brain activity. The studies have revealed that motor processing is often linked to beta oscillatory activity (Espenhahn et al., 2019; Schmidt et al., 2019). Learning requires plasticity and it is the balance between inhibitory and excitatory process in the brain reflected in the amplitude of frequencies, especial beta frequency (14–30 Hz) in the sensorimotor cortex (Espenhahn et al., 2019), and prefrontal cortex (PFC; Schmidt et al., 2019). In PFC, increased beta appeared at the end of the trial when working memory information needs to be erased (Schmidt et al., 2019). It was found that high CIE (i.e., random practice) results in a balance between inhibition and excitation networks in the brain (Chalavi et al., 2018). Furthermore, studies have shown that there is an optimal level of cognitive processing for optimal motor learning (Kahneman, 1973; Lin et al., 2008). Cognitive processing is related to alpha band (8–12 Hz) in the frontal lobe. The reduced alpha in frontal lobe reveals high cognitive processing and vice versa (Kropotov, 2010). However, it has been shown that random practice results in a high activation at prefrontal (e.g., DLPFC) and motor (e.g., M1) cortices (Lage et al., 2015), other studies have revealed that cognitive style and individual differences may influence EEG frequencies during information processing (Riding et al., 1997). According to the challenge point framework, individual differences in CIE can be covered by the interaction between skill level and task difficulty (Guadagnoli and Lee, 2004). Therefore, practice based on an optimal challenge point (i.e., algorithm practice) may affect EEG frequencies and motor outcomes. Although the neurological basis of contextual interference has been studied in previous research (Henz et al., 2018; for a review see Lage et al., 2015), it is hard to find a study that addressed the brain activity in algorithm-based practice in older adults.

Two different theories attempt to describe the beneficiary high CIE: the reconstruction action plan hypothesis (forgetting-reconstructing) and the elaborative hypothesis (distinctive and meaningful processing). Concerning the first hypothesis, random practice results in forgetting and reconstruction of the action plan in working memory (Lee and Magill, 1985). The second hypothesis states that random practice results in more distinctive and deeper meaningful processing (Shea and Zimny, 1983). It appears that the emergence of these mechanisms in a practice session is different. The reconstruction of the action plan and elaborative hypotheses are formed at the first and the last practice session, respectively (Magill and Hall, 1990; Yuhua, 1994; Boutin and Blandin, 2010a). In studies conducted by Lin et al. (2008, 2010), the neural mechanisms involved in CIE were examined through transcranial magnetic stimulation (TMS) during the trial intervals. The result demonstrated that the random practice group with TMS utilized at the motor area showed performance decrement during the retention test. In another research study conducted by Cohen et al. (2009), the CIE was examined during the movement preparation using TMS. The result indicated that random practice plays a role only in movement preparation, but not in motor execution. In their study, Frömer et al. (2016) examined the effect of event-related potential (ERP) on motor preparation of CIE. The results indicated that although cognitive processing related to attention increased in high CIE during the early acquisition, the cognitive and motor processes were severely declined in delayed retention. Although the processing related to the CIE associated with the motor preparation and intertrial interval periods has been investigated in the previous studies (Lin et al., 2008, 2010; Cohen et al., 2009; Frömer et al., 2016), in the current study, we used a new practice schedule (i.e., algorithm-based practice) and evaluated the processing in a between- within the design. It is possible to evaluate the reconstruction of the action plan hypothesis (forgetting-reconstructing) and also the elaborative hypothesis in a more concise manner by using the EEG method.

Briefly, despite the neural examinations to investigate the CIE, earlier studies were not carefully designed to examine this effect based on their distinctive cognitive mechanisms for the motor preparation and intertrial interval (motor evaluation) periods. For the first time, this study was designed to compare the neurological mechanisms of both reconstruction and elaborative processing hypotheses separately, using a similarity variable. Also, to date, no study has investigated the effect of practice based on the challenge point (i.e., algorithm practice) on EEG frequencies and we examined a novel practice schedule based on the individual cognitive style on EEG bands, especially alpha and beta bands that are related to cognitive and motor processing (Kropotov, 2010; Espenhahn et al., 2019; Schmidt et al., 2019). Besides, this study adopted a between—within-group design to perform the statistical analysis in comparison with the analysis applied in the previous studies, which only used either a within-group design (Cohen et al., 2009; Wymbs and Grafton, 2009) or a between-group design (Lin et al., 2008, 2010). Therefore, the purpose of this study was to investigate the neurological foundation underlying CIE and parameter similarity on motor learning in older adults.

## Materials and Methods

### Participants

Sixty healthy older adults (mean age, 67.68 ± 3.95 years; all men), right-handed (Edinburgh inventory; Oldfield, 1971), were enrolled in this study. Participants were randomly divided into six groups of blocked-similar (BS), algorithm-similar (AS), random-similar (RS), blocked-dissimilar (BD), algorithm-dissimilar (AD), or random-dissimilar (RD). Exclusion criteria were neurological disease (e.g., Parkinson), dementia [e.g., Alzheimer’s disease, cognitive mild impairment; according to Montreal of cognitive assessment (MoCA with cut-off <26; Nasreddine et al., 2005)], uncorrected vision and inadequate sleep (sleep timing questionnaire; Monk et al., 2003). The written informed consent was obtained from all participants. They had no previous experience at the task and were unaware of the purpose of this study. The study protocol was approved by the Ethics Committee of Ferdowsi University of Mashhad (Code: IR.UM.REC.1397.013).

### Apparatus and Procedure

#### Behavioral Section

**Figure 1:**
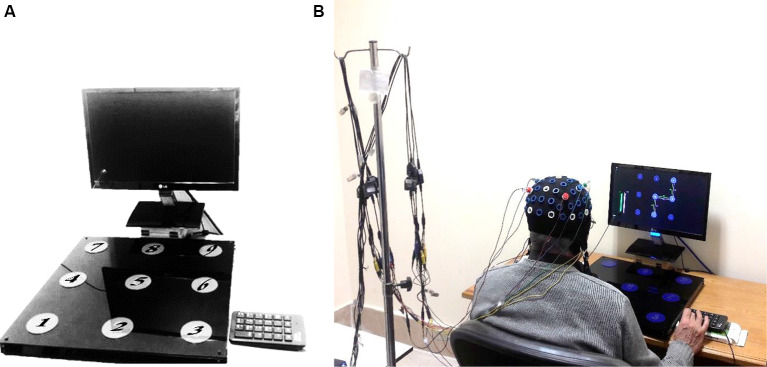
Sequential timing device **(A)**, and procedure of experiment in progress **(B)**.

The device used in this study was similar to that used by Boutin and Blandin (2010a). Hardware included a foam board 50 × 50 cm that contained nine keys with 6.5 cm diameter and 10 cm apart from each other (Figure 1A). The task was sequentially depressing the 2, 5, 6, 9 keys with the dominant (right) hand in Absolute Timing Goals (ATGs). ATGs were defined as the amount of time elapsed from depressing the first key (number two) to the last key (number nine). Participants had to perform three ATGs (1,350, 1,500, 1,650 ms) in a similar parameter and three ATGs (1,050, 1,500, 1,950 ms) in the dissimilar parameter. The 1,500 ms ATG was the mean of performance for the older adults based on a pilot study. The similar and dissimilar conditions were defined as a ±10% and ±30% difference from the mean ATG (1,500 ms), respectively (Boutin and Blandin, 2010a). A LabView-based (LabView, National Instrument 2018, Austin, TX, USA) custom software program was used for displaying the patterns, recording the responses, and switching the ATGs.

**Figure 2:**
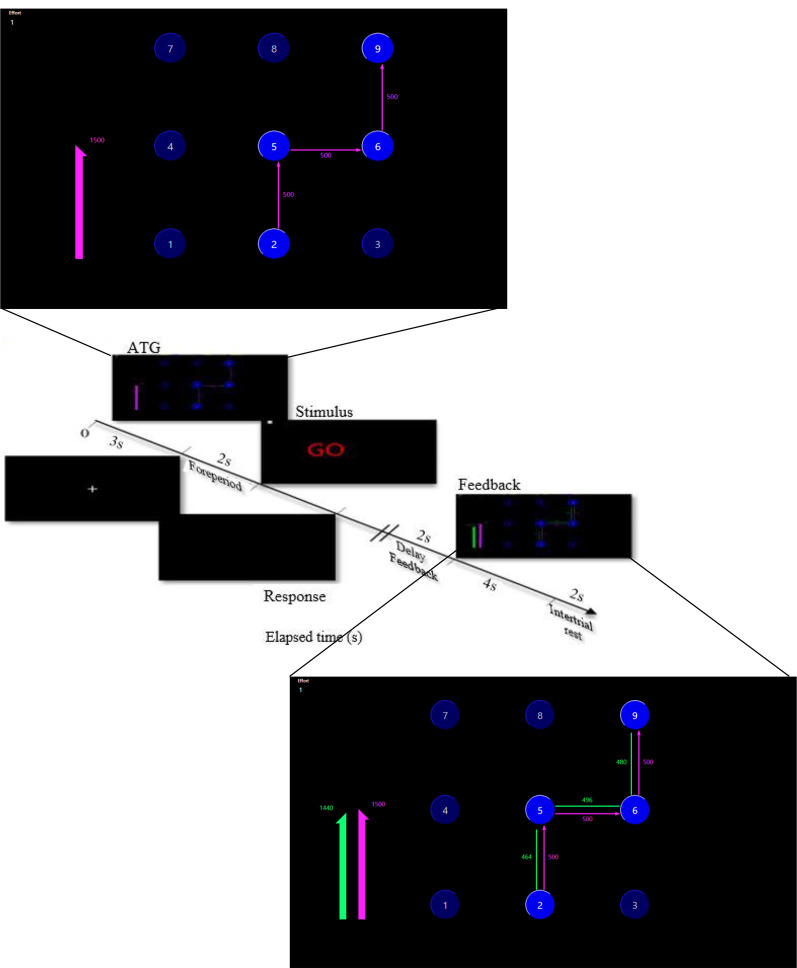
Procedure of one trial.

Participants performed all the experimental stages individually in a quiet room at the Motor Behaviour Laboratory. Every performer sat on an adjustable chair behind a table in front of the device and a monitor (Figure 1B). Every trial was started by releasing the Enter key of the numeric pad beside the device (Figure 1A). During the acquisition phase, participants performed 162 trials in 18 blocks (nine trials per block) and received delayed visual knowledge of results (KR) after accomplishing each trial. The procedure for performing one trial is presented in Figure 2. Each block was interspaced by 4 s rest intervals. Between every six-block of trials, there were 3 min rest to prevent fatigue. The participants were instructed to maintain the same time interval to move for pressing two consecutive keys to keep the relative timing fixed. Furthermore, the performers were encouraged to try their best performance in each trial as closely as to the ATGs. In a condition that participants missed a key, an error message was shown and that trial was immediately repeated.

**Figure 3:**
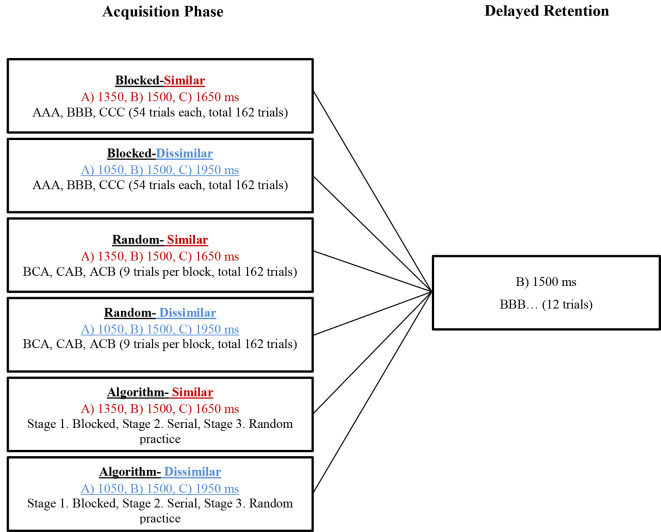
The paradigm of the experimental groups in the acquisition phase and delayed retention. Note that algorithm practice was a combination of blocked, serial, and random practice that forward and/or not backward switching between stages was based on error rate (number of error trial ≤33% in each block based on error range of the ATGs, ±5%).

**Figure 4:**
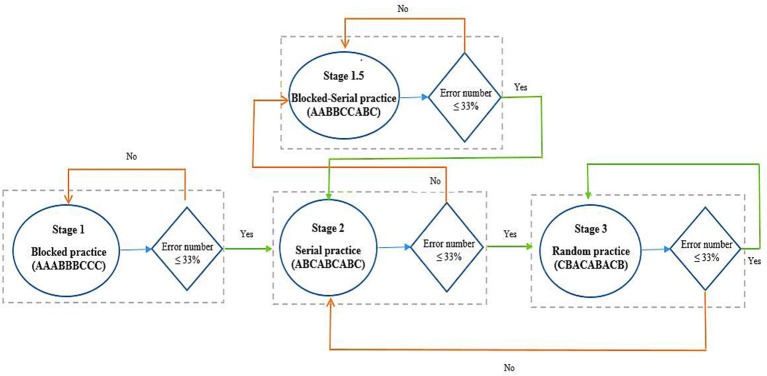
Computer algorithm and how to change the stages in the algorithm practice schedule. Follow the arrows in regards to Yes/No.

During the acquisition, all participants practiced the tasks (ATGs) according to their grouping (see Figure 3). The blocked practice groups (in the similar and dissimilar conditions) performed 54 trials in each ATG (total 162 trials) in a blocked order (AAA, BBB, CCC), distributed in six blocks of nine trials. The order of practice of the ATGs was counterbalanced between the participants. The random practice groups (in the similar and dissimilar conditions) performed 18 blocks of nine trials in random order in each block with the constraints that each block included three trials of each ATG and no ATG was practiced in two consecutive trials (e.g., BCA, CAB, ACB). The algorithm practice was a combination of blocked, serial, and random practice schedules. The algorithm practice groups (in both of similarity conditions) were switched between blocked, serial- and random-practice orders (known as stages 1, 2, and 3, respectively) in forward and/or not backward (progression and/or not regression) concerning the number of error trial limitations in each block (≤33%) and based on ATGs error range (±5%; Table 1). The algorithm practice was designed to maintain the level of functional task difficulty between a moderate to a high range. A trial was considered as an error if the difference between the performed timing with the ATG of that trial (the ideal time) was higher than ±5 percent. For example, the error range for 1,500 ms ATG was between 1,425 and 1,575 ms, and if the performed timing was outside of this range (e.g., 1,420 or 1,580 ms), that trial was known as one error trial. The error limit (criterion level) was considered to be 33% of nine trials in each block. The procedure of algorithm practice was as follows. The participants started the practice with blocked order (the first block). At the end of the first block, if the number of error trials was equal or lower than three (33%), the blocked practice (stage 1) was switched to serial practice (stage 2) in the second block. If the number of error trials was higher than the criterion level (more than three trials), the first stage (i.e., blocked practice) was repeated. In the second stage, participants practiced the ATGs in the serial order and if the number of error trials was lower than—or equal to—the criterion, they moved to the third stage (i.e., random practice) in the next block. If their number of error trials was higher than the criterion, they moved to a stage that we called stage 1.5. In this stage, the practice order was blocked-serial (AABBCCABC) and this was to maintain the performers’ motivation. In stage 1.5, if the number of errors was lower than—or equal to—the criterion, the participants were moved to the second stage (i.e., serial practice) in the next block, and if the number of errors was higher than the criterion, this stage was repeated. The practice order in the third stage was random. If the number of error trials was lower than—or equal to—the criterion, this stage was repeated, and if the number of errors was higher than the criterion, they moved to the second stage (see schematic representation in Figure 4). Note that switching the stages was depending on participants’ performance (error rate at the end of each block) in the algorithm practice groups, therefore, task difficulty was individualized in regards to participant’ progress (i.e., individualized optimal challenge point).

**Table 1:** The error range of absolute timing goals (ATGs) in algorithm practice schedule groups.

Group	Error range				
	1,350 ms (±67.5)	1,500 ms (±75)	1,650 ms (±82.5)	1,050 ms (±52.5)	1,950 ms (±97.5)
Algorithm-Similar	1,282.5–1,417.5 ms	1,425–1,575 ms	1,567.5–1,732.5 ms	*	*
Algorithm-Dissimilar	*	1,425–1,575 ms	*	997.5–1,102.5 ms	1,852.5–2,047.5 ms

*Lack of existence that ATG in algorithm similar/dissimilar groups.

A delayed retention test was performed 24 h after the acquisition. During the retention, 12 trials with no-KR were performed. The mean ATG (1,500 ms) was used as the task during delayed retention (Figure 3).

#### EEG Recording

EEG brain activity was recorded by the 10-channel FlexComp differential amplifier and Biograph software (Version 5.0.3) developed by Thought Technology (TT) of Canada. Eight channels were used to record the EEG signals according to the international 10-20 system, placed on the scalp with two reference electrodes to the ipsilateral ears of the active electrode and two ground electrodes to the contralateral ears of the active electrode. One channel was also used for the TT AV-Sync sensor to determine the beginning of each section of a trial on the EEG signals. EEG data were documented in the Biograph database and processed using a custom script written in MATLAB (Version R2017b, MathWorks, USA). To collect signals, first, the scalp area was carefully scrubbed with NuPrep abrasive gel, and then the electrodes were pasted by utilizing Ten20 paste. Impedances of all electrodes were kept below 5 kΩ in all trials. The signals were amplified with a time constant of 2 s (high pass filter: 0.1 Hz; low pass filter: 64 Hz). Furthermore, a 50 Hz notch filter (for line noise) was enabled. EEG signals were continuously recorded and digitized at a sampling rate of 256 Hz. Absolute power was calculated for alpha (8–12 Hz) and beta (14–30 Hz) bands in frontal (Fp1, Fp2, F3, F4), central (C3, C4), and parietal (P3, P4) cortices in the acquisition phase and delayed retention test.

### Data Analyses

#### Behavioral Analysis

Data analysis in the acquisition phase was performed by applying a three-way analysis of variance (ANOVA), 3 (practice schedule: blocked, algorithm, random) × 2 (similarity: similar, dissimilar) × 18 (blocks: 1–18) with repeated measures on the last factor. Data analysis in delay retention test were also performed by univariate ANOVA, 3 (blocked, algorithm, random) × 2 (similar, dissimilar).

#### EEG Analysis

Data analysis in the acquisition phase were performed using three-way ANOVA, 3 (practice schedule: blocked, algorithm, random) × 2 (similarity: similar, dissimilar) × 8 (scalp location: Fp1, Fp2, F3, F4, C3, C4, P3, P4) with repeated measures on the last factor. Two periods of motor preparation and intertrial interval have been considered in data analysis during the acquisition phase, separately. The first and last six blocks of the acquisition was used to analyze the EEG signals in motor preparation and intertrial interval periods, respectively. The signals were analyzed during 2 s before movement execution in motor preparation period (Cohen et al., 2009; Frömer et al., 2016), and analyzed during 2 s immediately after the feedback in the intertrial interval period (Lin et al., 2008, 2010). Data analysis in the delayed retention test was similar to the acquisition phase performed using mixed-design ANOVA, 3 × 2 × 8 with repeated measures on the last factor in the motor preparation period.

According to the normality of data (Shapiro-Wilk test) and homogeneity of variance (Levene’s test), we used the parametric test for the behavioral and EEG data (ANOVA with repeated measures). Also, Mauchly’s test of sphericity was not violated, except for the main effect of the block in behavioral data that Greenhouse–Geisser correction was used. Bonferroni *post hoc* test was used to determine the means differences between the groups for both behavioral and EEG analyses. The significance level for all analyses was set at *p* ≤ 0.05 using SPSS version 25 (SPSS Inc., Chicago, IL, USA).

## Results

### Behavioral

#### Total Error (E)

##### Acquisition Phase

**Figure 5:**
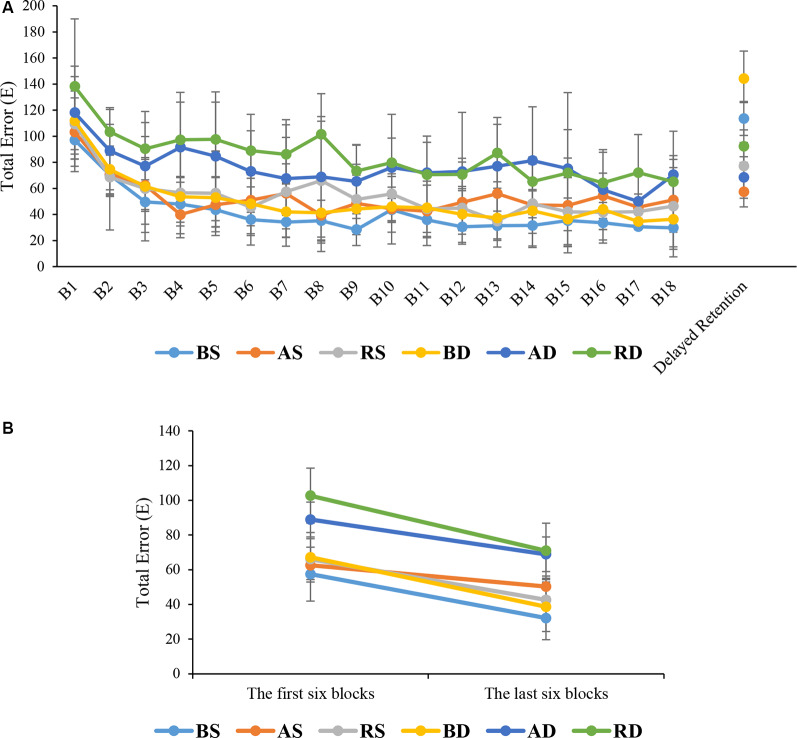
Means and standard deviations of the total error of the groups in **(A)** different phases and **(B)** the first and last six blocks (for a direct comparison with EEG data).

The ANOVA results showed that there were significant main effects for practice schedule (*F*_(2,54)_ = 21.645, *p* < 0.001, $\eta_{\text{p}}^{2}$ = 0.445), similarity (*F*_(1,54)_ = 43.299, *p* < 0.001, $\eta_{\text{p}}^{2}$ = 0.445), and block (*F*_(10.73,579.58)_ = 22.827, *p* < 0.001, $\eta_{\text{p}}^{2}$ = 0.297). In addition, there was a significant interaction effect of practice schedule × similarity (*F*_(2,54)_ = 4.435, *p* = 0.016, $\eta_{\text{p}}^{2}$ = 0.141). No significant interaction was found between the block and other factors (all *F* < 1). *Post hoc* test for interaction effect showed that there were significant differences between the RD and AD groups compared to BD, BS, AS, and RS groups (all *p* < 0.001), but no significant difference was found between the RD and AD groups (*p* > 0.05). Also, there was no significant differences among BS, AS, RS, and BD groups (all *p* > 0.05). A comparison of means showed that RD group had more error than other groups (means: RD = 84.77, AD = 76.09, BD = 49.64, RS = 54.02, AS = 53.17, BS = 41.41; see Figure 5A).

##### Delayed Retention

The results of ANOVA showed that the main effects of practice schedule (*F*_(2,54)_ = 63.552, *p* < 0.001, $\eta_{\text{p}}^{2}$ = 0.702), similarity (*F*_(1,54)_ = 15.077, *p* < 0.001, $\eta_{\text{p}}^{2}$ = 0.218), were significant but the interaction effect of practice schedule × similarity (*F*_(2,54)_ = 1.477, *p* = 0.237, $\eta_{\text{p}}^{2}$ = 0.052), were not significant. The *post hoc* test for the main effect of practice schedule showed that there were significant differences between the algorithm practice compared with blocked and random practice schedules (*p* < 0.001 and *p* = 0.002, respectively). As can be seen in Figure 5A, a comparison of means showed that the algorithm practice had lower error than blocked and random practice (means: algorithm = 62.88 ms, random = 84.79 ms, and blocked = 128.90 ms). Also, the mean comparisons showed that similar conditions had lower error than dissimilar conditions (means: similar = 82.73 ms, dissimilar = 101.65 ms).

### EEG

#### Acquisition Phase

##### Motor Preparation Period

**Figure 6:**
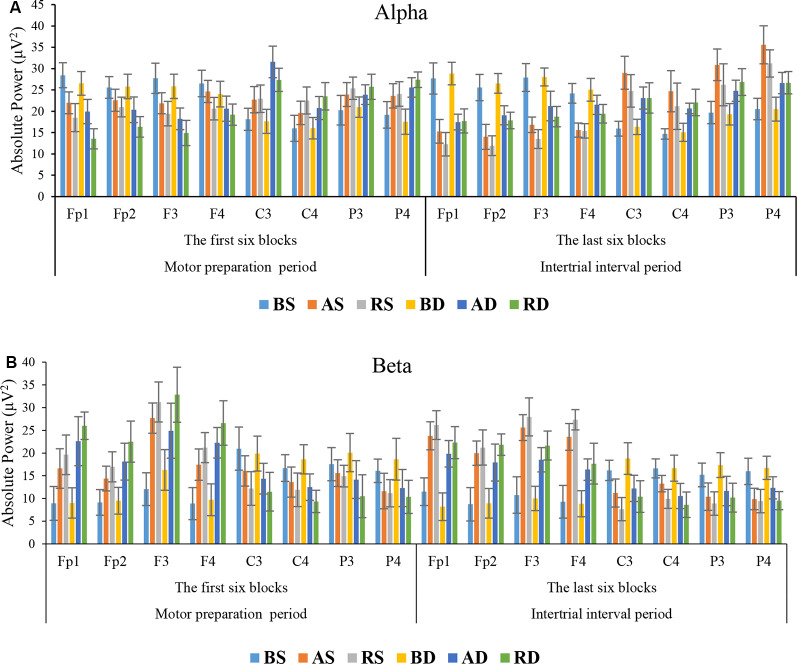
Means and standard deviations power of the groups in **(A)** alpha band and **(B)** beta band in different scalp locations at the acquisition phase.

Alpha Band: the results of ANOVA showed that there were significant main effects of practice schedule (*F*_(2,54)_ = 7.855, *p* = 0.001, $\eta_{\text{p}}^{2}$ = 0.225), similarity (*F*_(1,54)_ = 5.023, *p* = 0.029, $\eta_{\text{p}}^{2}$ = 0.085), location (*F*_(7,378)_ = 11.457, *p* < 0.001, $\eta_{\text{p}}^{2}$ = 0.175), and interaction effects of location × practice schedule (*F*_(14,378)_ = 47.455, *p* < 0.001, $\eta_{\text{p}}^{2}$ = 0.637), location × similarity (*F*_(7,378)_ = 13.383, *p* < 0.001, $\eta_{\text{p}}^{2}$ = 0.199), and location × practice schedule × similarity (*F*_(14,378)_ = 3.536, *p* < 0.001, $\eta_{\text{p}}^{2}$ = 0.116), but there was no significant effect of practice schedule × similarity (*F*_(2,54)_ = 1.263, *p* = 0.291, $\eta_{\text{p}}^{2}$ = 0.045). *Post hoc* test of interaction effect of location × practice schedule × similarity revealed that there were significant differences between the RD group and other groups in frontal lobe (all *p* < 0.001). *Post hoc* test for interaction also indicated that there were significant differences between the AD compared to other groups in C3 (all *p* < 0.05). As shown in Figure 6A, the mean comparisons of frontal lobe showed that the RD had less activity than other groups (means: RD = 16.01, AD = 19.78, RS = 19.89, AS = 22.77, BD = 25.56, and BS = 27.07). Also, the mean comparisons of C3 area showed that the AD group had more activity than other groups (means: AD = 31.58, RD = 27.36, RS = 22.94, AS = 22.71, BS = 18.14, and BD = 17.65).

Beta Band: the results of ANOVA showed that there were significant main effects of the practice schedule (*F*_(2,54)_ = 60.240, *p* < 0.001, $\eta_{\text{p}}^{2}$ = 0.691), similarity (*F*_(1,54)_ = 20.800, *p* < 0.001, $\eta_{\text{p}}^{2}$ = 0.278), location (*F*_(7,378)_ = 44.645, *p* < 0.001, $\eta_{\text{p}}^{2}$ = 0.453), and interaction effects of location × practice schedule (*F*_(14,378)_ = 45.273, *p* < 0.001, $\eta_{\text{p}}^{2}$ = 0.626), location × similarity (*F*_(7,378)_ = 4.566, *p* < 0.001, $\eta_{\text{p}}^{2}$ = 0.078), and location × practice schedule × similarity (*F*_(14,378)_ = 2.678, *p* = 0.001, $\eta_{\text{p}}^{2}$ = 0.090), but no significant effect was observed for practice schedule × similarity *F* < 1. *Post hoc* test for the interaction effect for the location × practice schedule × similarity revealed that there were significant differences among the RD group and other groups in frontal lobe (all *p* < 0.001). As can be seen in Figure 6B, the mean comparisons showed that the RD group had more activity than other groups (means: RD = 26.99, AD = 21.98, RS = 22.25, AS = 19.03, BD = 11.12, and BS = 9.75).

##### Intertrial Interval Period

Alpha Band: the results of ANOVA showed that there were significant main effects of practice schedule (*F*_(2,54)_ = 9.442, *p* < 0.001, $\eta_{\text{p}}^{2}$ = 0.259), location (*F*_(7,378)_ = 60.985, *p* < 0.001, $\eta_{\text{p}}^{2}$ = 0.530), and interaction effects of practice schedule × similarity (*F*_(2,54)_ = 5.303, *p* = 0.008, $\eta_{\text{p}}^{2}$ = 0.164), location × practice schedule (*F*_(14,378)_ = 91.204, *p* < 0.001, $\eta_{\text{p}}^{2}$ = 0.772), location × similarity (*F*_(7,378)_ = 22.667, *p* < 0.001, $\eta_{\text{p}}^{2}$ = 0.296), and location × practice schedule × similarity (*F*_(14,378)_ = 5.899, *p* < 0.001, $\eta_{\text{p}}^{2}$ = 0.179), but there was no significant main effect of similarity (*F*_(1,54)_ = 1.851, *p* = 0.179, $\eta_{\text{p}}^{2}$ = 0.033). *Post hoc* test for interaction effect of location × practice schedule × similarity revealed that there were significant differences among the RS group and BS, BD, AD, and RD groups in frontal lobe (all *p* < 0.001), but no significant difference was found between RS and AS groups (*p* = 0.069). *Post hoc* test for interaction effect also indicated that the AS group performed significantly different from the other groups in C3 (all *p* < 0.05) and P4 (all *p* < 0.05). As shown in Figure 6A, comparison of the mean values in the frontal lobe showed that the RS group had less activity than other groups (means: RS = 13.28, AS = 15.43, RD = 18.45, AD = 19.82, BS = 26.35, and BD = 27.12).

Beta Band: the results of ANOVA showed that there were significant main effects of practice schedule (*F*_(2,54)_ = 48.740, *p* < 0.001, $\eta_{\text{p}}^{2}$ = 0.644), similarity (*F*_(1,54)_ = 22.490, *p* < 0.001, $\eta_{\text{p}}^{2}$ = 0.294), location (*F*_(7,378)_ = 60.577, *p* < 0.001, $\eta_{\text{p}}^{2}$ = 0.529), and interaction effects of the practice schedule × similarity (*F*_(2,54)_ = 7.075, *p* = 0.002, $\eta_{\text{p}}^{2}$ = 0.208), location × practice schedule (*F*_(14, 378)_ = 80.347, *p* < 0.001, $\eta_{\text{p}}^{2}$ = 0.748), location × similarity (*F*_(7,378)_ = 15.538, *p* < 0.001, $\eta_{\text{p}}^{2}$ = 0.223), and location × practice schedule × similarity (*F*_(14,378)_ = 2.187, *p* = 0.008, $\eta_{\text{p}}^{2}$ = 0.075). *Post hoc* test for interaction effect of location × practice schedule × similarity revealed that there were significant differences among the RS group and other groups in frontal lobe (all *p* < 0.05). The mean comparisons showed that the RS group had more activity than other groups (means: RS = 25.66, AS = 23.23, RD = 20.86, AD = 18.17, BS = 10.08, and BD = 9.01; see Figure 6B).

##### Delayed Retention

**Figure 7:**
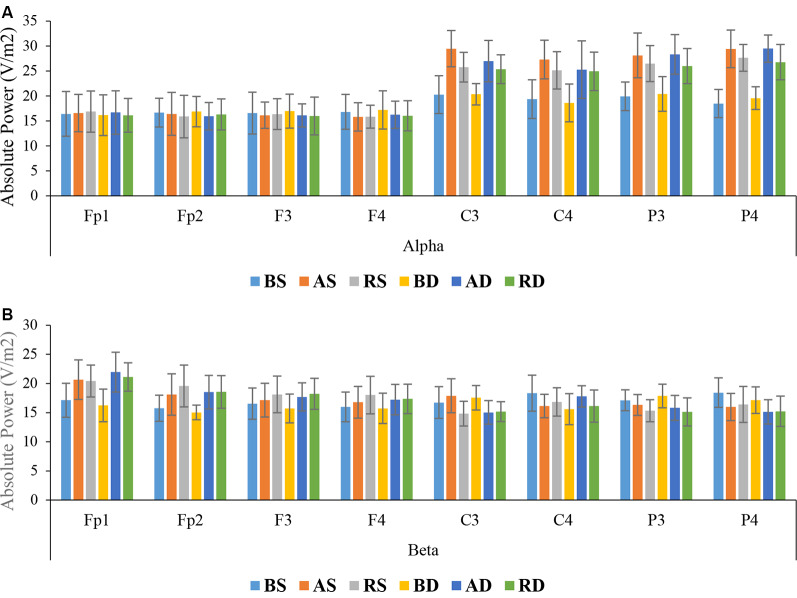
Means and standard deviations power of the groups for **(A)** alpha band and **(B)** beta band in different scalp locations in the delayed retention test.

Alpha Band: the results of ANOVA showed that there were significant main effects of practice schedule (*F*_(2,54)_ = 73.454, *p* < 0.001, $\eta_{\text{p}}^{2}$ = 0.731), location (*F*_(7, 378)_ = 164.346, *p* < 0.001, $\eta_{\text{p}}^{2}$ = 0.753), and interaction effect of location × practice schedule (*F*_(14,378)_ = 18.566, *p* < 0.001, $\eta_{\text{p}}^{2}$ = 0.407), but there were no significant effects of similarity, and interaction of location × similarity, location × practice schedule × similarity, and practice schedule × similarity, all *F* < 1. *Post hoc* test for interaction effect of location × practice schedule revealed that there were significant differences between the algorithm groups and other groups at C3 (*p* < 0.001 and *p* = 0.035, respectively) and P4 (*p* < 0.001 and *p* = 0.034, respectively). The mean comparisons showed that the algorithm groups had more activity than other groups (means at C3: algorithm = 28.22, random = 25.55, blocked = 20.29; means at P4: algorithm = 29.44, random = 27.20, blocked = 19.01; see Figure 7A).

Beta Band: the results of ANOVA showed significant main effects of practice schedule (*F*_(2,54)_ = 3.471, *p* = 0.038, $\eta_{\text{p}}^{2}$ = 0.114), location (*F*_(7,378)_ = 11.025, *p* < 0.001, $\eta_{\text{p}}^{2}$ = 0.170), and interaction effects of location × practice schedule (*F*_(14,378)_ = 6.398, *p* < 0.001, $\eta_{\text{p}}^{2}$ = 0.192), but the main effects of similarity (*F*_(1,54)_ = 1.183, *p* = 0.182, $\eta_{\text{p}}^{2}$ = 0.033), and interaction effects of practice schedule × similarity location × similarity all *F* < 1, and location × practice schedule × similarity were not significant (*F*_(14,378)_ = 1.177, *p* = 0.291, $\eta_{\text{p}}^{2}$ = 0.042). The *post hoc* test for interaction effect of location × practice schedule revealed significant differences between the random and algorithm practice as compared with the blocked practice in frontal lobe (all *p* < 0.001), but no significant difference was found between the random and algorithm practice schedules (*p* > 0.05). The mean comparisons indicated that the random groups had more activity than other groups (means: random = 18.92, algorithm = 18.51, blocked = 16.02; see Figure 7B).

## Discussion

The purpose of this study was to investigate the neural mechanisms of CIE and parameter similarity on motor learning in older adults. The results of this study confirmed the previous findings of other studies demonstrating that high CI (random practice) can increase the activities of cognitive, sensory, and motor regions of the brain compared to blocked practice (Cross et al., 2007; Lin et al., 2008, 2010, 2013; Cohen et al., 2009; Wymbs and Grafton, 2009; Thürer et al., 2017; Henz et al., 2018). The older adults also benefit from the high CI in reducing their error during the retention test and also their cognitive, sensory, and motor areas activated more than blocked practice. In their study, Henz et al. (2018) demonstrated that random practice increased beta wave in the frontal lobe. Other studies applied TMS or fMRI reported that random practice can increase DLPFC—an area related to the cognitive processing and motor preparation—and M1 activity (for a review see Lage et al., 2015).

The results of this study indicated that algorithm practice as an optimal level of error led to an optimal level of activation in the frontal lobe (medium levels of alpha and beta activities) in motor preparation and intertrial interval (motor evaluation) periods of performing a motor task. This optimal level of activation in the frontal resulted in a maximized of sensory and motor excitation (an increase of alpha wave in parietal and central regions). The trend of the first and last six blocks for total error showed that in similar and dissimilar conditions, the algorithm practice had moderate error than other groups. These results are comparable with EEG results showing moderate alpha and beta waves in the frontal area during algorithm practice compared with other practice schedules (see Figures 5B, 6A,B). Also, the total error decreased in the last six blocks in the intertrial interval and EEG results revealed the RS had a moderate activity of alpha and beta in the frontal area and maximum activity of alpha in C3 and P4 compared with the other groups. In the first six blocks in motor preparation periods, the RD had a moderate error and moderate alpha and beta activation and maximum activation of alpha at C3 (see Figures 5B, 6A,B). Cognitive control of motor processing is directly associated with the increase of beta and reduction of alpha waves in the frontal lobe (Kropotov, 2010; Henz et al., 2018). Also, the increase in the alpha wave in parietal and central regions is an indicator of an increase in sensory integration and motor memory (Kanayama et al., 2015; Henz et al., 2018). In PFC, the assessment of beta band in motor preparation and intertrial interval periods showed that the random practice resulted in the highest beta activity and the algorithm based practice resulted in a moderate beta activity while the blocked practice resulted in the lowest beta activity in this area. Studies have shown that increased beta in PFC is related to the working memory process of movement. It is suggested that this increase is an index of erased working memory (Lundqvist et al., 2018; Schmidt et al., 2019). Therefore, even though an increase in cognitive effort results in the improvement of memory coding and decoding, the studies have demonstrated that there is a desirable level of attention allocation and cognitive processes based on the task difficulties and skill level of the learner for the optimal motor learning output (Kahneman, 1973; Guadagnoli and Lee, 2004; Lin et al., 2008).

**Figure 8:**
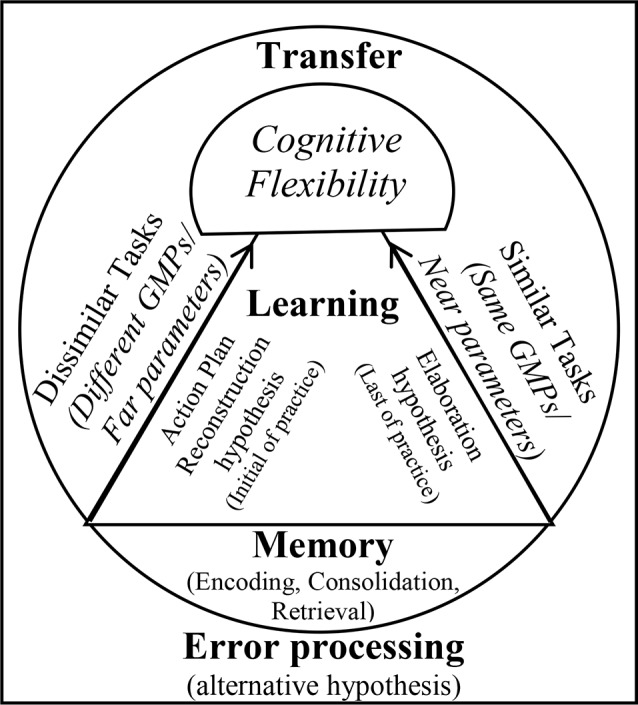
Proposed Contextual Interference Model (CIM).

The findings obtained from the brain waves confirm the cognitive flexibility (Berry et al., 2016), cognitive load (Sweller et al., 2019), and schema (Schmidt, 1975) theories about the CIE. Our results indicated that algorithm practice optimized alpha and beta band in frontal and maximized alpha band in sensorimotor regions (at C3 and P4). The absolute power of alpha and beta in frontal areas for algorithm schedule was between the random (the highest power) and blocked (the lowest power) schedules. Also, the alpha power in sensorimotor regions (C3 and P4) was maximized during the algorithm practice compared with other schedules. These EEG results should be considered along with the error data. The results of behavioral data showed that during acquisition, the algorithm schedules resulted in an error range between the blocked (the lowest error range) and the random (the highest error range) schedules. This type of brain activation and this range of error during the algorithm schedule resulted in the lowest error range during the retention test. According to these results one can conclude that during the algorithm practice an optimal level of cognitive processing occurred, resulted in optimal motor learning during retention (Kahneman, 1973; Guadagnoli and Lee, 2004; Lin et al., 2008). Cognitive flexibility in aging is related to frontoparietal connectivity (Berry et al., 2016), and increased beta and alpha bands reflect plasticity in memory (Espenhahn et al., 2019). Lin et al. (2013) found that interleaved practice enhances skill learning due to increased frontoparietal networks. According to cognitive load theory, working memory has a limited capacity for information processing (Sweller et al., 2019). Possibly, the algorithm practice with providing optimal cognitive processing facilitated optimal cognitive load in the frontal lobe (medium alpha and beta waves). Also, the findings of this study revealed that in similar conditions, beta activity increased in frontal, and alpha activity increased in central and parietal lobes at the end of acquisition, while in dissimilar conditions, beta activity enhanced in frontal, and alpha activity enhanced in central and parietal lobes at the beginning of acquisition. According to the schema theory, there are two types of memory involved in learning a motor task: the recall memory that is in charge in producing the movement and the recognition memory that is in charge of evaluating the movement (Schmidt, 1975). The distinction between the reconstruction and elaborative processing hypotheses based on the neural mechanism can be explained by the views they adopt. Based on our findings, both mechanisms of reconstruction and elaboration emerged in different stages of practice. Both of these are cognitive processes: the reconstruction hypothesis during motor preparation and before the performance recalls the movement memory (reinforcing the recall memory) and elaborative hypothesis is in charge for evaluation between the course of trials and comparing inter-trial performances (reinforcement of recognition memory). In a study conducted by Lin et al. (2008, 2010) the TMS was applied during the interval between trials to impair M1 region in different practice arrangements. The results confirmed strongly the elaborative hypothesis due to TMS-Random practice condition, however, the reconstruction hypothesis was not supported because of the lack of enhancement of learning in TMS-Blocked practice condition. Accordingly, in other studies, TMS was applied to impair M1 during the movement preparation period and the result indicated that random practice condition showed performance decrement during the delayed retention test and consolidation of performance was detected in No-TMS and Sham-TMS groups (Cohen et al., 2009; Wymbs and Grafton, 2009). The difference in performances may be attributed to two mechanisms that can improve memory as follows. One is the space effect and the other is CIE. Therefore, it appears that contextual interference has more instability effect on memory compared to the space effect and increases the long retention and generalizability (Robertson, 2018). Blocked-TMS groups have experienced space effect to reconstruct the action plan in the M1 region, while the Random-TMS group results in interference effect for different action plans. Therefore, it may be plausible to explain why the reconstruction hypothesis of Lin et al. (2008, 2010) was less supported. The difference between space and interference effects may be due to the memory formations. Consequently, the researcher proposed the conceptual model for the CIE (Figure 8).

Also, two paths for motor memory encoding and retrieval in motor tasks have been discussed in the literature review, indicating the frontal to central and parietal to central pathways (Verwey et al., 2015). In other words, the excitation of the motor region takes place through two pathways. This study showed that algorithm practice optimized these pathways with an optimal activation of alpha and beta waves in frontal, and high activation of motor area (high alpha at central region) that these changes could be related to motor memory encoding (Lin et al., 2013; Henz et al., 2018). Also, the results showed that algorithm practice resulted in the activation of sensorimotor regions (high alpha power in central and parietal) that could be related to motor memory retrieval (Lin et al., 2013; Henz et al., 2018). Furthermore, our results indicated that the interaction effect of practice arrangement and similarity provides a different neurological mechanism. At the beginning of the random practice group, the highest levels of activities occurred in the frontal lobe (increase in beta power) and the algorithm-dissimilar group had the highest level of activity in the motor area (an increase of alpha power), however, at the end of practice, the random-similar group had the highest level of activity in the frontal lobe and algorithm-similar group exhibited the highest level of activity in the sensory and motor regions. In fact, in similar conditions, most of the parietal and frontal regions during the intertrial interval period were activated through the elaboration mechanism at the end of acquisition (the last six-block), resulting in a maximum level of activation in motor regions (high alpha at C3 and P4). However, in the dissimilar conditions, most of activities in frontal lobe led to the maximum level of activity of alpha band in contralateral motor region (C3) throughout reconstructing the action plan during the early phase of acquisition processes (the first six-block).

The results obtained from EEG data in this study confirmed the findings of previous studies demonstrating that restructuring action plan develops at the early stage of acquisition, while the elaborative hypothesis develops at the last stage of acquisition (Yuhua, 1994; Brady, 1998; Boutin and Blandin, 2010a), and the practice amount affects this process (Shea et al., 1990). In similar conditions, the EEG results showed that the high activation of the beta band in frontal increased at the end of the acquisition. In similar conditions due to close ATGs, the elaborative mechanism was needed. While, in dissimilar conditions due to far ATGs, high activation of beta in frontal increased at the early of acquisition (reconstructive mechanism). According to the schema theory, the parameterization of movement occurs during the practice process as a whole. However, according to the parameter remapping phenomenon, with the extension of practice, parameterization occurs separately (Rosenbaum et al., 1986; Verwey et al., 2015). Accordingly, this may be the reason for introducing a generalized parameter for similar parameters to each other in the early practice (similar conditions). EEG studies have shown that absolute timing is specified before muscle group/effector (Leuthold and Jentzsch, 2011) and absolute force (Shea and Wulf, 2005).

Also, the results of this study showed that contralateral motor region was more activated (high alpha at C3) during performing the task with the dominant (right) hand which were consistent with the findings of previous studies suggesting that motor regions were more activated in the contralateral limb of performers (alpha wave at C3; Lin et al., 2008, 2010). However, other studies reported that more activities took place in the motor region of the ipsilateral limb (Cohen et al., 2009; Wymbs and Grafton, 2009). One of the reasons for these inconsistent findings may be related to the use of dominant/non-dominant hand in these studies during the task execution, while all of the participants in these inconsistent studies were right-handed. Therefore, despite the contradictory findings in terms of the increase of activity in the ipsilateral or contralateral limb, the similarity of these studies is associated with the increase in activity of the left hemisphere of the brain and right-handedness of performers. The results of some studies have indicated that the left hemisphere of motor cortex is dominant in motor learning, particularly when the non-dominant hand is utilized, and the complexity of the movement increases when the non-dominant hand is used (Kawashima et al., 1993; Ziemann and Hallett, 2001; Suzuki et al., 2013).

Studies have shown that practice under the high CI when impairing the M1 region using TMS immediately after the acquisition phase resulted in a decrement of performance in a practice session. After one night of rest, it did not affect retention test, while disturbance using TMS before the acquisition phase resulted in performance decrement in delayed retention test even after a night sleep (Wymbs and Grafton, 2009). Therefore, it appears that high CI results in more flexible neural traces that can facilitate coding, storing, and retrieval and make motor-memory less vulnerable to interference and time passing. However, the behavioral and neural results of our study indicated that the optimal level of interference based on a performance algorithm and optimal error rate resulted in the highest efficiency in performance and brain activity.

## Conclusion

This study showed that algorithm-based practice resulted in the performance enhancement of motor learning in elderly learners. Also, our findings demonstrated that algorithm-based practice led to an optimal activity in sensorimotor areas due to optimal cognitive processes involvement. Alpha and beta bands are related to motor performance (Espenhahn et al., 2019; Schmidt et al., 2019). This study showed that algorithm practice optimized alpha and beta band in frontal (as cognitive processing center) and maximized activation of sensorimotor regions (at C3 and P4). However, the random practice showed higher activation of alpha and beta bands in the prefrontal lobe, but evidence has revealed that optimal activation of cognitive processing results in optimal motor learning (Kahneman, 1973; Guadagnoli and Lee, 2004; Lin et al., 2008) Furthermore, EEG result revealed that task similarity affects the activation of beta at early or late of the acquisition. In dissimilar conditions, increased beta activity in frontal lobe was observed at the early of acquisition, while in similar conditions, it was seen at the late of acquisition. Future studies need to investigate the rehabilitation and clinical applications of this type of practice to increase the efficiency of the practice.

## Data Availability Statement

The datasets generated for this study are available on request to the corresponding author.

## Ethics Statement

The studies involving human participants were reviewed and approved by Ethics Committee of Ferdowsi University of Mashhad (code: IR.UM.REC.1397.013). The patients/participants provided their written informed consent to participate in this study.

## Author Contributions

MB, HT, and AS designed the experiment, and MB performed the experiment. All authors contributed to analyze the data and MB wrote the manuscript. All authors approved the final version of the manuscript.

## Conflict of Interest

The authors declare that the research was conducted in the absence of any commercial or financial relationships that could be construed as a potential conflict of interest.
